# Chronic short sleep duration lengthens reaction time, but the deficit is not associated with motor preparation

**DOI:** 10.1111/jsr.14231

**Published:** 2024-05-23

**Authors:** Caroline Dutil, Julia De Pieri, Christin M. Sadler, Dana Maslovat, Jean‐Philippe Chaput, Anthony N. Carlsen

**Affiliations:** ^1^ School of Human Kinetics, Faculty of Health Sciences University of Ottawa Ottawa Ontario Canada; ^2^ Healthy Active Living and Obesity Research Group Children's Hospital of Eastern Ontario Research Institute Ottawa Ontario Canada; ^3^ School of Epidemiology and Public Health, Faculty of Medicine University of Ottawa Ottawa Ontario Canada

**Keywords:** alertness, chronic sleep duration, cognitive performance, reaction time, StartReact effect

## Abstract

The purpose of this study was to investigate the association between chronic sleep duration and reaction time performance and motor preparation during a simple reaction time task with a startling acoustic stimulus in adults. This cross‐sectional study included self‐reported short sleepers (*n* = 25, ≤ 6 hr per night) and adequate sleepers (*n* = 25, ≥ 7.5 hr per night) who performed a simple reaction time task requiring a targeted ballistic wrist extension in response to either a control‐tone (80 dB) or a startling acoustic stimulus (120 dB). Outcome measures included reaction times for each stimulus (overall and for each trial block), lapses, and proportion of startle responses. Chronic short sleepers slept on average 5.7 hr per night in the previous month, which was 2.8 hr per night less than the adequate sleepers. Results revealed an interaction between sleep duration group and stimulus type; the short sleepers had significantly slower control‐tone reaction times compared with adequate sleepers, but there was no significant difference in reaction time between groups for the startling acoustic stimulus. Further investigation showed that chronic short sleepers had significantly slower control‐tone reaction times after two blocks of trials lasting about 5 min, until the end of the task. Lapses were not significantly different between groups. Chronic short sleep duration was associated with poorer performance; however, these reaction time deficits cannot be attributed to motor preparation, as startling acoustic stimulus reaction times were not different between sleep duration groups. While time‐on‐task performance decrements were associated with chronic sleep duration, alertness was not. Sleeping less than the recommended sleep duration on a regular basis is associated with poorer cognitive performance, which becomes evident after 5 min.

## 1 INTRODUCTION

Numerous factors have been shown to modulate reaction time (RT) in apparently healthy individuals, including factors related to stimulus processing, such as stimulus type (Galton, 1899), intensity (Piéron, 1920; Wells, 1913) or number of possible stimuli (Hick, 1952), as well as factors related to response execution, such as task complexity (Henry & Rogers, 1960; Klapp, 2010), stimulus–response compatibility (Fitts & Deininger, 1954; Fitts & Seeger, 1953), mental acuity (Schweizer, 2001), age (Myerson et al., 2007) and the amount of sleep a person gets (Cote et al., 2009; Goel et al., 2009; Lim & Dinges, 2010; Van Den Berg & Neely, 2006). Sleep deprivation has been shown to have a strong influence on alertness and cognitive performance, affecting RT. Research, including the seminal work of Patrick and Gilbert published at the end of the 19th century (Patrick & Gilbert, 1896), highlighted a linear dose–response relationship in neurobehavioural deficits including slower RT, that was associated with up to 90 hr of sleep deprivation. More recently, a large meta‐analysis of 70 studies (147 datasets) found that short‐term sleep deprivation (19–48 hr) negatively affects cognitive domains like processing speed, complex attention, and working memory, with the most pronounced impact on simple attention, which showed the largest effect size (Lim & Dinges, 2010). Nonetheless, decrements in RT due to sleep deprivation for other domains such as processing speed, complex attention, and working memory also showed compelling moderate effect sizes despite substantial heterogeneity between the tasks (Lim & Dinges, 2010). These findings not only highlight the continued interest in understanding the role of sleep on cognitive performance, but more importantly emphasize the importance of sleep and its impact across many cognitive domains. While most studies focus on acute sleep deprivation or restriction, chronic sleep restriction, a more common form of sleep loss, remains relatively understudied.

Chronic sleep restriction is typically characterized as adults consistently receiving less than the recommended 7–8 hr of sleep per night (Chaput et al., 2020). In Canada and the USA, approximately one‐third of adults do not meet the recommendations for minimum sleep duration per night (Chaput et al., 2017; Sheehan et al., 2019). Therefore, the first objective of this study was to investigate the association between *chronic* sleep duration and RT during a simple auditory RT task. This was accomplished by comparing RT in a sample of apparently healthy adults who were classified as chronic short sleepers (i.e. people who in the past month averaged a sleep duration that does not meet the sleep guidelines), versus adults who were classified as chronic adequate sleepers (i.e. people who in the past month averaged a sleep duration that meets the sleep guidelines).

As noted above, the negative effect of sleep deprivation on RT is often linked to deficits in alertness and time‐on‐task deficits (Mackworth, 1964). The Psychomotor Vigilance Task (PVT) is commonly used to measure these deficits, as tracking response latencies and errors over a 10‐min period (Basner & Dinges, 2011; Dinges & Powell, 1985). However, the PVT does not assess preparedness for movement, leaving the influence of sleep duration on motor preparation processes unclear. One novel method to gauge preparation level is through the presentation of a startling acoustic stimulus (SAS) during a RT task. Research has shown that a prepared voluntary movement can be involuntarily triggered by a SAS at a very short latency (Carlsen et al., 2004a; Valls‐Solé et al., 1995; Valls‐Solé et al., 1999; Valls‐Solé et al., 2008) when a startle reflex (Landis & Hunt, 1939) is elicited – a phenomenon known as the StartReact effect. Importantly, this effect occurs only when the movement is prepared in advance and when there are enough cognitive resources to maintain a high preparation level (Carlsen et al., 2004a; Drummond et al., 2013; Maslovat et al., 2015; Peters et al., 2022; Valls‐Solé et al., 1999). Additionally, the StartReact effect is characterized by a high proportion of startle reflexes measurable via electromyography (EMG) over the sternocleidomastoid (SCM), and it does not exhibit quick habituation when a movement has been prepared (Blumenthal et al., 2005; Brown et al., 1991; Carlsen et al., 2003; Carlsen et al., 2004a).

Therefore, the second objective of the study was to utilize a SAS during a simple RT task in order to perform a comparative analysis of motor preparation level between chronic sleep duration groups (short versus adequate). It was hypothesized that if motor preparation deficits are associated with slowed RT in participants with chronic short sleep duration, both the SAS trials and control trial would be slower and the proportion of SAS trials with startle reflexes would be lower, compared with adequate sleepers. On the other hand, if RT on SAS trials and the proportion of SAS trials with startle reflexes are not significantly different between chronic short sleepers and adequate sleepers, then RT deficits would not be associated with motor preparation processes.

## 2 METHODS

### 2.1 Participants, exposure and comparator

The study was conducted and reported in accordance with the guidelines set forth by the Declaration of Helsinki, the ethical guidelines set by the University of Ottawa's Research Ethics Board, and the Strengthening the Reporting of Observational Studies in Epidemiology (STROBE) statement (Von Elm et al., 2007). Written informed consent was obtained from all participants. A convenience sample of apparently healthy adults was recruited at the University of Ottawa, Canada, between December 2018 and March 2019 to participate in this cross‐sectional study.

To be eligible to participate, apparently healthy adults needed to be either chronic short duration sleepers (defined as self‐reporting sleeping ≤ 6 hr per night on average in the previous month) or chronic adequate duration sleepers (defined as self‐reporting sleeping ≥ 7.5 hr per night on average in the previous month). Sleep duration was subjectively assessed using the Pittsburgh Sleep Quality Index (PSQI; Buysse et al., 1989). Additionally, in order to ensure that the night prior to testing was not atypical of their average sleep reported on the PSQI, participants also completed a short questionnaire asking about their previous night of sleep (i.e. bedtime, wake time, and length of time to fall asleep). If the night prior to testing was deemed atypical (i.e. > ± 30 min) of their monthly average sleep, participants were ineligible to participate due to sleep variability as a possible confounder. No participants were excluded due to sleep variability. Participants were instructed to not consume any caffeine in the 2 hr preceding their testing session, as caffeine intake prior to testing may be a confounder. For those drinking coffee every morning, upon waking up, we made sure to schedule testing later in the day, disturbing daily routine and regular caffeine intake may also be a confounder. In addition to meeting the sleep duration group criteria to be eligible, participants were also required to have normal or adjusted to normal vision and hearing, to be fluent in English, and to have no upper right limb physical disorder or disability.

Based on previously reported effect sizes (Lim & Dinges, 2010), we determined that a total sample size of 46 (23 per group) would be sufficient for the present study, as it would provide 80% power at 0.05 level of significance (two‐sided) to detect a 20% increase in RT in chronic short duration sleepers, which is a conservative increase in RT compared with acute sleep deprivation or restriction studies. Based on our sample size calculation, we decided to recruit two additional participants per group, to account for possible missing data. For this study, the exposure group was 25 chronic short duration sleepers and the comparator group was 25 chronic adequate duration sleepers. Although this was a convenience sample, everyone that was approached to participate in the study agreed.

### 2.2 Experimental set‐up

**FIGURE 1:**
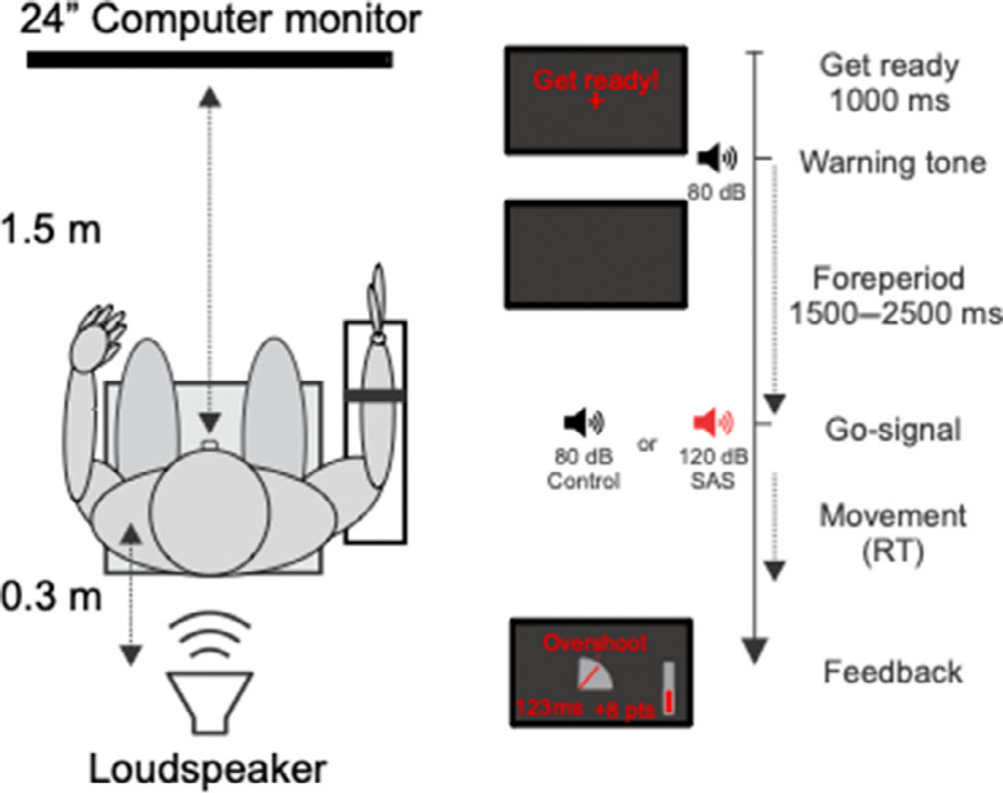
Schematic overhead view of participant set‐up (left) and trial timing (right) for the simple reaction time (RT) task using a startling acoustic stimulus (SAS). Note trial timing represents the temporal sequence of events of a single trial, and illustrates visual prompts, stimuli, and feedback presented.

Participants were seated comfortably in front of a computer monitor set at eye‐level ~1.5 m away. Their right arm rested in a custom‐made manipulandum that placed their limb in ~30 degrees of shoulder abduction, 90 degrees of elbow flexion, and with their wrist in a semi‐prone position so that their palm faced inwards. In order to isolate movement but not restrict the range of motion of the wrist joint, the participant's forearm was secured in the manipulandum armrest by a Velcro strap. A speaker was placed behind the participant's head, levelled with the ears at a distance of 30 cm. In order to minimize white and blue light exposure during testing, the task was conducted in a darkened room with the feedback display monitor set to night colours (Figure 1).

### 2.3 Experimental procedures

The experimental task was a simple RT task that required participants to make a targeted wrist extension movement with the right hand in response to an auditory imperative stimulus. The control‐tone consisted of a briefly presented single tone (1000 Hz, 100 ms duration, 80 ± 2 dB), and the SAS involved a brief intense white noise burst (25 ms duration, 120 ± 2 dB, < 1 ms rise time). The experiment included six blocks of 30 trials (including SAS trials), for a total of 180 experimental trials. The first two blocks contained no SAS trials, whereas in blocks 3–6, the ratio of SAS to control‐tone trials was increased incrementally as follows: block 3: 1:15; block 4: 1:10; block 5: 1:6; block 6: 1:5. The number of SAS exposures for all participants was 16 of the 180 total trials. SAS trials were pseudo‐randomly interspersed (i.e. first two trials of a block could not be a SAS trial, and there were never two consecutive SAS trials) within their respective blocks. Prior to commencing the task, participants performed five familiarization trials that were not analysed.

To start each trial, participants were provided with a representation of their current wrist position as well as the location of the target for 1 s along with the words “Get Ready” on the computer screen. After this, the online feedback was removed, a warning tone (200 Hz, 100 ms duration, 80 ± 2 dB) was presented, and the screen was fully darkened. Following the warning tone, a variable foreperiod of 1500–2500 ms preceded the second auditory tone, which served as the “Go” signal (Figure 1). Following the auditory warning tone and display of “Get Ready” on the monitor, participants were instructed to prepare a right‐handed 20 degree wrist extension movement from the starting position, and respond by releasing the prepared movement as quickly as possible when the second auditory tone was played. After each trial, terminal feedback was provided on the monitor for 3500 ms, which included displacement RT, proximity to the target (i.e. good, overshoot for movement > 25°, or undershoot for movement < 15°), and points awarded. To maintain motivation and task engagement, a cumulative point system was used in which participants received points for RTs on trials that were deemed fast (< 140 ms), and penalized for trials that were considered too slow (> 250 ms) or when participants anticipated and moved before the “Go” signal.

### 2.4 Outcome measures

The primary outcome measure for cognitive performance was premotor RT in the right extensor carpi radialis, measured as the time between stimulus presentation and EMG onset of the muscle. EMG onset was defined as an increase in the rectified and filtered signal exceeding two standard deviations from the baseline activation (mean of 100 ms of EMG prior to the Go‐signal). Additionally, we also looked at the effect of time‐on‐task on RT performance. Time‐on‐task was defined as the observed RT performance during each of the trial block (blocks 1–6 for control‐tone and blocks 3–6 for SAS tone) between the two chronic sleep duration groups. For time‐on‐task, it was decided that it would only be investigated if we found performance differences between the chronic sleep duration groups in the main analysis. Alertness was measured in terms of the number of lapses in the task and proportion of SAS trials with startle reflexes. Lapses were defined as trials where the participant had slow responses (RT > 500 ms), omissions and movement errors. For each participant, the proportion of SAS trials with a startle reflex was calculated as the number of SAS trials where an EMG burst was noted in SCM within 120 ms of the SAS (Carlsen et al., 2011), divided by the total number of SAS trials presented. For motor preparation, we used premotor RT in the right extensor carpi radialis in conjunction with EMG from the left SCM during the SAS trials. During the SAS trials, SCM was used as startle reflex indicator, and trials with no visible SCM activation were not used in the analyses as they are not indicative of high levels of preparation. Four participants’ (two adequate and two short durations sleepers) SAS trials data were removed from the analyses as they did not startle.

### 2.5 Recording equipment

Surface EMG was collected from the superficial muscle bellies of the right extensor carpi radialis (agonist), right flexor carpi radialis (antagonist) and left SCM (as an indicator of a startle reflex) using bipolar, pre‐amplified parallel‐bar electrodes (Delsys Bagnoli DE‐2.1) connected via shielded cabling to an external amplifier (Delsys Bagnoli‐8). To minimize electrical impedance, all electrode attachment sites were cleaned with abrasive skin gel (NuPrep) and alcohol wipes prior to attachment. The electrodes were aligned parallel to the muscle fibres and attached to the skin using adhesive double‐sided tape. Finally, a reference electrode (Dermatrode HE‐R) was placed on the right medial epicondyle. Unfiltered EMG were digitally sampled at 4000 Hz using a custom LabVIEW program and stored for offline analysis. Data collection was initiated by the computer on each trial beginning 1000 ms prior to presentation of the auditory Go‐signal and continued for 3000 ms.

### 2.6 Statistical analysis

Descriptive characteristics of the sample are presented for each group (chronic short sleepers versus chronic adequate sleepers), as well as for all participants overall. For normally distributed continuous variables, mean and standard deviation are reported, whereas *n* and percentage are reported for categorical variables. Data analyses were performed using SPSS statistical software (Version 29, SPSS, Chicago, IL, USA) and JASP (Version 0.18.3, JASP Team, Netherlands). All variables were examined for normality using the Shapiro–Wilk test, and no variables needed transformation. An independent *t*‐test was performed to confirm a significant difference in sleep duration between the self‐reported chronic short sleepers and chronic adequate duration sleepers. To ascertain the association between chronic sleep state, performance (RTs) and motor preparation (RT following the SAS), we performed a mixed‐model analysis of variance (ANOVA) comparing the interaction between the two chronic sleep duration groups (short versus adequate sleepers) and the two Go‐signals (control versus SAS). It was decided a priori that if a significant difference was found between chronic state groups and RT in response to one or both Go‐signals, then secondary analyses using mixed‐model ANOVAs would be used to determine the association between chronic sleep state and time‐on‐task (six blocks for regular Go and four blocks for startle Go). Additionally, for the post hoc analyses for the association between chronic sleep groups and time‐on‐task, it was also predetermined that we would only explore between group differences for each block of trials individually using Bonferroni correction (six comparisons for the control‐tone, and four comparisons for the SAS tone). To determine the association between chronic sleep state and alertness, we conducted independent *t*‐tests comparing lapses and the proportion of SAS trials with startle reflexes between chronic sleep duration groups. In cases where the assumption of sphericity was violated, Greenhouse–Geisser‐corrected degrees of freedom were used to report the main effect. Finally, based on feedback from the peer‐review process, we included two new analyses response variability and reciprocal response time (Basner & Dinges, 2011; Dinges et al., 1987). For each participant, standard deviations and reciprocal response time for each tone were calculated and analysed in the same manner as previously described for RT. Significant differences with a probability of less than 0.05 were considered significant. Significant interactions were decomposed using independent and paired *t*‐tests with corrections for multiple comparisons using the Holm–Bonferroni method. Cohen's *d* or partial eta‐squared (*η*_p_^2^) are reported where appropriate to provide an estimate of effect size (Cohen, 1988). Variations of the effects were verified for time of testing, age, education level, use of sleep medication and biological sex.

## 3 RESULTS

### 3.1 Descriptive characteristics

An independent *t*‐test showed a significant difference in hours of self‐reported sleep duration between our chronic short and adequate sleeper groups (*t*_48_ = 6.2, *p* < 0.001, *d* = 0.68), with a sleep duration difference between the two groups of 2 hr 47 min per night on average in the previous month (Table 1).

**TABLE 1:** Descriptive characteristics of the sample by chronic sleep duration group and overall.

Variable	Chronic short sleepers (*n* = 25)	Chronic adequate sleepers (*n* = 25)	Total (*n* = 50)
Age in years	24.0 (4.2)*	21.8 (3.2)*	22.8 (3.8)
Self‐reported sex, *n* (%)			
Female	16 (64)	15 (60)	31 (62)
Male	9 (36)	10 (40)	19 (38)
Self‐reported handedness, *n* (%)			
Right‐handed	24 (96)	25 (100)	49 (98)
Left‐handed	1 (4)	0 (0)	1 (2)
Sleep duration (hr per night in the past month)	5.7 (0.7)*	8.5 (0.6)*	7.1 (1.6)
Mean total PSQI score	7.9 (3.2)*	4.2 (1.8)*	6.0 (2.9)
PSQI sleep efficiency percentage	91.3 (8.2)*	96.5 (2.3)*	93.9 (6.5)
Use of sleep medication, *n* (%)			
Not in the past month	22 (88)	20 (80)	42 (84)
Less than once a week	3 (12)	3 (12)	6 (12)
Once or twice a week	0 (0)	0 (0)	0 (0)
Three or more times a week	0 (0)	2 (8)	2 (4)
PSQI interpretation, *n* (%)			
Good sleep quality	5 (20)	17 (68)	22 (44)
Poor sleep quality	20 (80)	8 (32)	28 (56)
Sleep satisfaction percentage	44.2 (18.8)*	69.6 (16.2)*	56.9 (21.6)
Time of day of testing, *n* (%)			
Morning	8 (32)	8 (32)	16 (32)
Midday	8 (32)	6 (24)	14 (28)
Late afternoon	9 (36)	11 (44)	20 (40)

*Note*: Values are presented as means with standard deviations (SD) in brackets for normally distributed continuous variables, and *n* and percentage for categorical variables. A chronic short sleeper was defined as self‐reporting an average sleep duration of ≤ 6 hr of sleep per night in the past month, while a chronic adequate sleeper was defined as self‐reporting an average sleep duration of ≥ 7.5 hr of sleep per night in the previous month. A higher sleep satisfaction percentage means a more positive feeling towards the last night of sleep before testing. Good sleep quality represents a total PSQI score < 5, and a poor sleep quality represents a total PSQI score > 4.

Abbreviation: PSQI, Pittsburgh Sleep Quality Index.

* Significant differences between chronic sleep duration groups were observed (*p* < 0.05).

### 3.2 Outcome measurement

**FIGURE 2:**
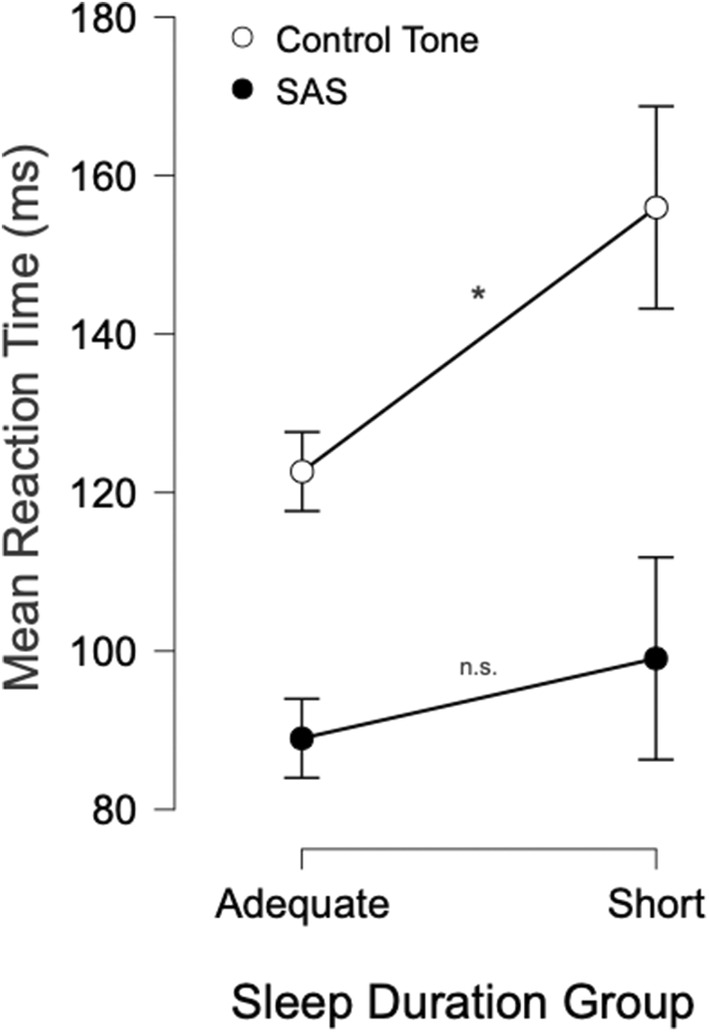
The association between chronic sleep duration group and mean premotor reaction time (RT) during a simple auditory RT task using a startling acoustic stimulus (SAS). A chronic short sleeper was defined as an individual self‐reporting sleeping ≤ 6 hr per night in the previous month, while a chronic adequate duration sleeper self‐reported sleeping ≥ 7.5 hr per night. Results for this figure were obtained using mixed‐model ANOVA, two chronic sleep duration groups by two Go‐signals. Error bars represent 95% confidence interval for each mean. *p‐value < 0.05 for the interaction effect. ANOVA, analysis of variance; n.s., not significant.

Premotor RT observed for the two groups is shown in Figure 2. Analysis of mean premotor RT between sleep group and stimulus type showed a significant main effect of stimulus type (*F*_1,44_ = 86.15, *p* < 0.001, *η*_p_^2^ = 0.66), indicating that RT latencies observed following the SAS tone were significantly faster than the latencies following the control‐tone. In addition, there was a significant main effect of chronic sleep group (*F*_1,44_ = 5.25, *p* = 0.027, *η*_p_^2^ = 0.10). However, these main effects were superseded by a significant interaction between the factors (*F*_1,44_ = 5.97, *p* = 0.019, *η*_p_^2^ = 0.12). Post hoc analysis of this interaction revealed that mean control‐tone RT was significantly different between chronic sleep duration groups. Specifically, the adequate sleepers showed shorter latencies when responding to the control‐tone (122.7 ms) compared with the short sleepers (156.0 ms; *p* = 0.007, *d* = 0.68). However, there was no significant difference in RT observed between sleep groups in response to the SAS (*p* = 0.26, *d* = 0.34; Figure 2). As expected, there was also a significant effect of stimulus type for both groups whereby the RTs observed following the SAS were significantly shorter than those following the control‐tone (*p* < 0.001 for each paired *t*‐test). Furthermore, we observed no significant main effect of sleep groups on response variability (*F*_1,44_ = 1.72, *p* = 0.196, *η*_p_^2^ = 0.04) or reciprocal response time (*F*_1,44_ = 3.57, *p* = 0.07, *η*_p_^2^ = 0.08). However, there were significant differences between tones for response variability (*F*_1,44_ = 117.77, *p* < 0.001, *η*_p_^2^ = 0.73) and reciprocal response time (*F*_1,44_ = 94.72, *p* < 0.001, *η*_p_^2^ = 0.68). Specifically, response variability was greater for the control‐tone compared with the SAS tone, with a mean difference of 33.7 ms and 95% confidence interval between 27.46 and 39.98 ms. On the other hand, reciprocal response time was greater for the SAS tone compared with the control‐tone, with a mean difference of 3.18 and 95% confidence interval between 2.52 and 3.84.

**FIGURE 3:**
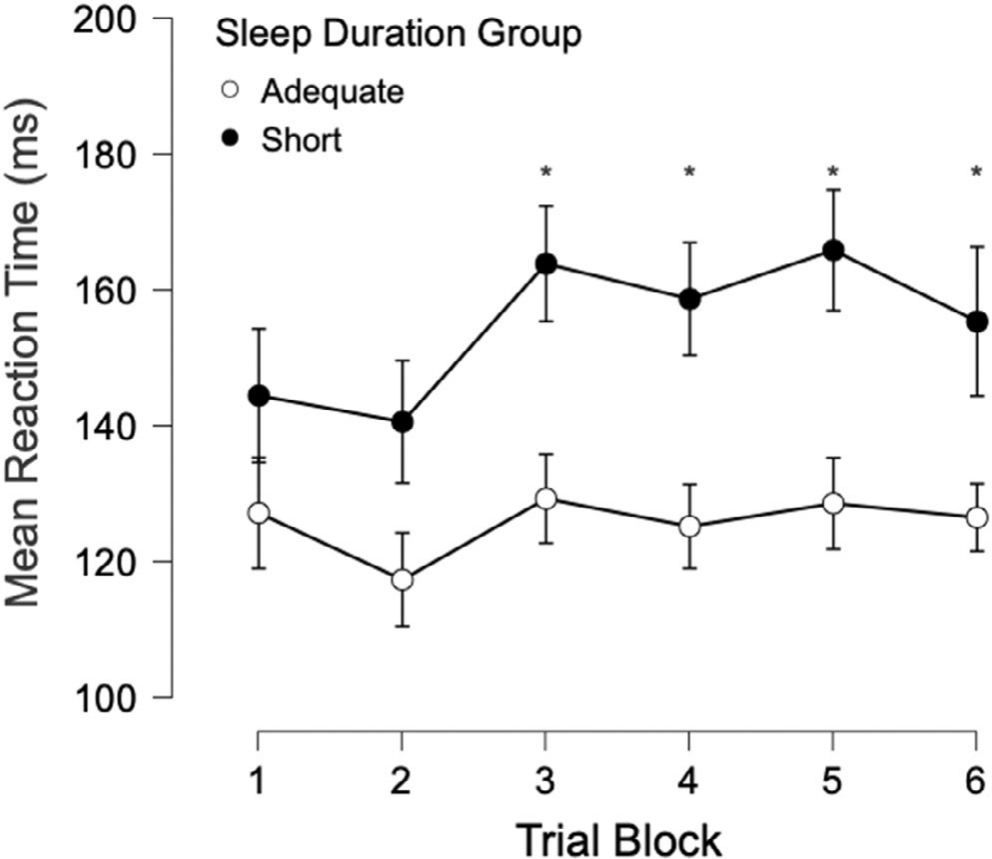
The association between chronic sleep duration group and mean premotor reaction time (RT) for the control‐tone as a function of time‐on‐task, represented by trial block, during a simple auditory RT task. A chronic short sleeper was defined as an individual self‐reporting sleeping ≤ 6 hr per night in the previous month, while a chronic adequate duration sleeper self‐reported sleeping ≥ 7.5 hr per night. Results were obtained using a mixed‐model ANOVA, two chronic sleep duration groups by six trial blocks. Error bars represent 95% confidence interval for each mean. *p‐value < 0.05 for the comparison between the chronic sleep duration groups and at each individual trial block. ANOVA, analysis of variance.

Secondary analysis investigating the association between chronic sleep duration group and RT performance to the control‐tone as a function of time‐on‐task was tested by comparing control‐tone RT as a function of trial block between the two sleep groups (Figure 3). Results revealed a significant effect of time (represented by the trial blocks) on control trial RT between the two sleep duration groups (Greenhouse–Geiser‐corrected degrees of freedom; epsilon = 0.786; *F*_3.91,88.8_ = 6.23, *p* < 0.001, *η*_p_^2^ = 0.12). There was also a significant main effect of chronic sleep group (*F*_1,48_ = 5.81, *p* = 0.020, *η*_p_^2^ = 0.11). Preplanned comparisons with Bonferroni correction of control‐tone RT between chronic sleep groups at each successive trial blocks revealed that the control‐tone RT for trials in blocks 1 and 2 were not significantly different between the two chronic sleep groups (*p* > 0.05); however, chronic short sleepers became significantly slower by approximately 26% (33.5 ms) in block 3 until the end of the task (blocks 3–6, all *p* < 0.05) compared with adequate sleepers.

The proportion of SAS trials where a startle reflex was observed in SCM was not significantly different between groups (*t*_48_ = 0.71, *p* = 0.48, *d* = 0.20). Analysis of the lapses also showed no significant difference between sleep groups (*t*_48_ = −0.14, *p* = 0.89, *d* = 0.04), in the mean number of lapses committed by adequate duration sleepers (16.6 lapses; SD = 8.4) versus short duration sleepers (16.9 lapses; SD = 10.2), representing an average error rate of ~9% for each group. No variations of the effect were found when taking age, level of education, biological sex, use of sleep medication or time of testing into account in the statistical models (all *p* > 0.4).

## 4 DISCUSSION

The purpose of this study was to investigate the association between chronic sleep duration, RT performance and motor preparation during a simple auditory RT task with a SAS. Overall, this study showed that chronic short sleepers, who did not meet the recommended sleep duration guidelines in the past month, slept on average 2 hr 47 min less per night in the previous month than the adequate sleepers, those who met the recommended sleep duration guidelines in the past month. Chronic short duration sleepers showed lengthened RT as compared with chronic adequate sleepers (Figure 2). However, this effect does not appear to be a result of a deficit in motor preparation as inferred from the lack of significant differences between the two chronic sleep duration groups for the SAS tone RT (Figure 2), variability, reciprocal response time and the proportion of startle reflexes. Additionally, we found no association between chronic sleep state groups and alertness, measured via lapses. Finally, time‐on‐task (as a function of trial block) was found to be associated with an increase in the control‐tone RT in chronic short sleepers compared with chronic adequate duration sleepers. Specifically, RT became significantly worse for the chronic short sleepers after completing two trial blocks, which represented only 5 min of actual work (Figure 3). Thus, the present study was not only able to investigate the association between chronic short sleep duration (a common form of sleep loss) and motor preparation, but was also able to quantify when time‐on‐task decrements occurred in this population.

According to the total PSQI results, five participants in the chronic short sleep duration group were considered good sleepers, while eight participants in the chronic adequate sleep duration group had poor sleep quality. The concept of sleep duration and adequacy is crucial for understanding the interplay between sleep and cognitive function. Sleep duration refers to the total amount of time an individual spends asleep, and there are known individual differences in this trait (Webb & Friel, 1971). These differences may or may not be linked to functional variations, such as cognitive performance (Aeschbach et al., 1996). On the other hand, sustained sleep restriction, where an individual consistently gets less sleep than they need, has been shown to impact on cognitive function, particularly RT. This effect was first demonstrated in two landmark studies from two decades ago, that tested groups that were assigned 3, 5, 7 or 9 hr of time in bed for 7 nights (Belenky et al., 2003), and 4, 6 or 8 hr of time in bed for 14 nights (Van Dongen et al., 2003). These studies underscored the negative effects of prolonged sleep restriction on performance, highlighting the critical role of sufficient sleep in maintaining optimal cognitive function. In the present study, we recruited based on public health sleep duration recommendations, recognizing that most individuals with chronic short sleep duration would likely be sleep restricted, but we also acknowledge that individual sleep needs may vary. While long sleepers are less common, they can indicate other sleep issues that are associated with their own health risks (Chaput et al., 2020). These findings underscore the importance of promoting adequate sleep beyond just sleep duration for cognitive function and overall health. Importantly, we did not exclude mismatched good and poor sleeper from our sleep groups analyses, as our chronic sleep duration comparators were determined a priori and excluding them would have been less representative of our study population.

As previously mentioned, very few studies have explored the role of sleep restriction on motor preparation. Two recent studies have investigated how total sleep deprivation affects motor preparation. Fournier et al. (2020) placed particular emphasis on the impact of sleep deprivation (38 hr of wakefulness) compared with well‐rested participants (10 hr of time in bed) on motor preparation during the coordination of multiple action plans. They found that sleep‐deprived participants exhibited significant difficulties in preparing and executing motor actions, especially when these actions were part of an overlapping action plan. Similarly, a study by Song et al. (2023) investigated the effects of total sleep deprivation (36 hr of wakefulness) on motor preparation sub‐stages during a visual search task. Using stimulus and response lateralized readiness potentials, they showed that total sleep deprivation impacted sensory integration but did not impact response execution (Song et al., 2023). Another study by Stojanoski et al. (2019) conducted a randomized crossover study to investigate the effects of mild acute sleep restriction (1 night of 5 hr of time in bed) compared with sleep extension (1 night of 9 hr of time in bed) on vigilance and cognitive processing. They measured indicators of reduced vigilance and perceptual and behavioural event‐related potentials during a sustained PVT over an extended daytime period (Stojanoski et al., 2019). The study found that mild acute sleep restriction negatively affected the processing of visual stimuli, as measured by visual‐evoked potentials, and reduced motor‐related responses following the presentation of the stimulus, as measured by lateralized readiness potentials (Stojanoski et al., 2019). These findings and ours highlight that total sleep deprivation, short‐term sleep and longer‐term sleep restriction impact cognitive and motor functions that are essential to activities of daily living.

Slower RT in simple RT task paradigms has been linked to reduced readiness for movement after the Go‐signal, possibly due to insufficient sleep. The two‐process model of sleep regulation, published 40 years ago and still relevant today in sleep research, describes the homeostatic process (Process S) that regulates sleep pressure, and the circadian process (Process C) that tracks time of day (Borbély, 1982). Results from neuroimaging suggest that the frontoparietal network is sensitive to Process S, while subcortical areas are sensitive to Process C (Muto et al., 2016). Our study found no RT variation based on testing time during the day, but we did not test throughout the 24‐hr period. Despite chronic sleep duration affecting control trial RT, no significant differences were observed in RT for SAS trials or in the proportions of startle responses between the chronic sleep duration groups. This suggests that a deficit in motor preparation processes does not explain the link between RT decline and chronic short sleep. The involuntary triggering of a prepared movement by a SAS likely involved a subcortical pathway, which is said to be less affected by lack of sleep (Carlsen et al., 2004a; Carlsen et al., 2004b; Muto et al., 2016; Valls‐Solé et al., 1999). The reticular activating system (RAS), known for regulating the sleep–wake cycle, fight‐or‐flight response and overall regulation of consciousness by a complex network that simultaneously connects the brainstem reticular formation all the way to the cerebral cortex, could be involved (Garcia‐Rill, 2015; Paus, 2000). From the perspective of the RAS in our study, a SAS, considered an intense stimulus, can simultaneously activate both the ascending and the descending RAS and result in not only the release of the prepared movement at reflex‐like latencies and a startle reflex, but also increase arousal at the cortical level. Because both response to the startle and the regulation of consciousness are mediated by the reticular formation, the lack of significant association between chronic sleep duration state and SAS trial RTs aligns with the notion that functioning in these neural pathways is preserved during bouts of sleep loss. While we have posited the two‐process model of sleep regulation as a possible explanation for our interaction findings, we also acknowledge that other paradigms may be relevant, such as top‐down and bottom‐up regulation or vigilance decrement and local, use‐dependent sleep. For a comprehensive review, see the work of Hudson et al. (2020).

In our study using a simple RT task with a warning signal before the Go‐signal, participants could prepare consistently their movement ahead of time, unlike the more random PVT. While measuring motor preparation is harder in the PVT due to its randomness and longer stimulus intervals, preparation is still necessary before movement. Our results, based on responses to SAS trials, suggest that lack of sleep is not linked to movement preparation processes. Therefore, the longer latencies seen in our task and other PVT studies likely result from other processing stages. Additionally, we expected that pre‐cueing participants to prepare for 1500–2500 ms would reduce differences in lapses between chronic sleep duration groups compared with a PVT. As expected, we found no significant differences in lapses between the groups, with both having an average error rate of 9%.

Although the chronic short sleep group's RT performance to the control‐tone was not consistently slower as compared with the chronic adequate sleep group, the short sleepers' performance significantly declined after only two trial blocks, which equated to 5 min of work. Specifically, the chronic short duration sleepers were, on average, ~35 ms slower during the final four blocks compared with adequate sleepers (Figure 3). Some neuroimaging studies suggest that sleep deprivation can lead to compensatory brain mechanisms that sustain performance despite the sleep loss (Drummond et al., 2001; Drummond et al., 2005; Drummond & Brown, 2001). To our knowledge, identifying the moment when RT degrades in chronic short duration sleepers is novel and could indicate when these compensatory mechanisms fail. Future research should further explore the effects of compensatory brain mechanisms associated with chronic short sleep.

If we consider lack of sleep as a stressor, there are several stress management responses and mechanisms that could also help explain the RT results. Briefly, during stress, the amygdala upregulates stress pathways in the hypothalamus and brainstem, leading to increased release of noradrenaline and dopamine (catecholamines; Arnsten, 2009). Under these circumstances, alertness regulation switches from a “top‐down” control by the prefrontal cortex to a more sensory‐driven “bottom‐up” control that can desensitize the prefrontal cortex, prioritizing attention to more salient or intense stimulus (such as the SAS tone; Arnsten, 2009; Buschman & Miller, 2007). The observed increase in RTs for chronic short duration sleepers also coincided with the introduction of the SAS tone in the task (trial blocks 3–6). Considering our study design and the results, it is possible that stress responses also play a role in the interaction we found between our chronic sleep duration groups and stimulus type. However, further investigation is needed to fully understand the relationships between chronic sleep duration, stress responses and startling stimuli.

### 4.1 Limitations

The findings of this study must be interpreted in the light of the following limitations. First, it is a cross‐sectional study, so we cannot establish causal relationship, and further experimental studies are needed to confirm our results. Additionally, our sample was a sample of convenience, which could introduce a risk of selection bias and limit the generalizability of our findings. While we measured sleep subjectively using a reliable and validated questionnaire, future studies should use objective measure for a more accurate assessment of sleep duration and other sleep characteristics (e.g. variability, fragmentation, objective efficiency, wake‐after‐sleep onset, architecture and chronotype). Some participants in our short duration sleep group might not have been habitual short sleepers, despite meeting the sleep duration criteria, as their lack of sleep might have been due to temporary factors like academic demands. The ramp protocol used for presenting the SAS trials aimed to assess our research question without causing an immediate arousal spike. However, the lack of significant differences in RT for the SAS trials between the two chronic sleep groups could be attributed to other unmeasured factors, potentially confounding the assessment of time‐on‐task effect. More research with different SAS protocols is needed to confirm these results. Because the result is null, it may be suggested that there was insufficient power to detect the significant difference in RTs for the SAS trials, although the study was not underpowered for other effects of the SAS, such as the proportion of startle reflexes. This implies that motor preparation is not associated with chronic sleep duration. While we did not observe any variation in the effect based on time of testing, it is plausible that the time of day could influence the findings. Ideally, in future studies, establishing a uniform testing time based on individual's waking habits would be preferable. Sleep needs vary among individuals, not everyone is affected equally by lack of sleep, and sleep quality is also an important factor to consider. Some participants in the chronic adequate sleep group had sleep durations higher than recommended, potentially due to unmeasured sleeping issues that could have impacted these results. Therefore, large sample sizes or a within‐subject experimental design would be preferable for such studies.

## 5 CONCLUSION

In summary, our data reveal that individuals with chronic short sleep duration (less than 6 hr per night in the past month) exhibited significantly impaired voluntary RTs compared with those with chronic adequate sleep. However, we did not find a link between effective motor preparation and chronic sleep duration, suggesting that the impact of chronic short sleep primarily affects other processes. Additionally, while alertness (measured by lapses and startle responses) was not associated with chronic short sleep duration, time‐on‐task was, as indicated by chronic short sleepers showing a 25% slower RT after only 5 min of work despite being well prepared. This suggests that compensatory brain mechanisms may have limited resources to maintain performance before deficits appear. The specific mechanism underlying this association remains unknown but warrants further investigation. Those consistently sleeping less than 6 hr per night may benefit from increasing their nightly sleep duration to sustain faster reactions during extended periods of focus work.

## AUTHOR CONTRIBUTIONS

**Caroline Dutil:** Conceptualization; methodology; data curation; formal analysis; project administration; writing – original draft; writing – review and editing; visualization. **Julia De Pieri:** Data curation; project administration; writing – review and editing. **Christin M. Sadler:** Writing – review and editing; visualization. **Dana Maslovat:** Methodology; conceptualization; software; writing – review and editing. **Jean‐Philippe Chaput:** Conceptualization; methodology; writing – review and editing; supervision. **Anthony N. Carlsen:** Conceptualization; methodology; software; funding acquisition; writing – review and editing; project administration; supervision; visualization; resources; data curation.

## FUNDING INFORMATION

This study was conducted at the University of Ottawa, and was funded by the Natural Sciences and Engineering Research Council of Canada funding agency (NSERC: RGPIN 2017‐04717).

## CONFLICT OF INTEREST STATEMENT

The authors declare that they have no conflicts of interest relevant to this article.

## Data Availability

The data that support the findings of this study are available from the corresponding author upon reasonable request.
